# The Combined and Single Effect of Marjoram Essential Oil, Ascorbic Acid, and Chitosan on Fresh-Cut Lettuce Preservation

**DOI:** 10.3390/foods10030575

**Published:** 2021-03-10

**Authors:** Panayiota Xylia, Antonios Chrysargyris, Nikolaos Tzortzakis

**Affiliations:** Department of Agricultural Sciences, Biotechnology and Food Science, Cyprus University of Technology, Limassol 3036, Cyprus; pa.xylia@edu.cut.ac.cy (P.X.); a.chrysargyris@cut.ac.cy (A.C.)

**Keywords:** minimally processed lettuce, *Origanum majorana* oil, ascorbic acid, chitosan, food quality, enzymes activity

## Abstract

Increasing demands by consumers for fresh, nutritional, and convenient food has led to the increase of fresh-cut produce market. Nowadays, there is a turn towards the investigation of natural products (i.e., essential oils, organic acids, and edible coatings) in an effort to lower the usage of chemical synthetic compounds (i.e., chlorine) as postharvest sanitizers. The aim of the present study was to assess the effectiveness of *Origanum majorana* essential oil (EO), ascorbic acid (AA), chitosan, and their combinations on quality attributes of fresh-cut lettuce stored for six days at 7 °C. When applied, Chitosan+AA resulted to a less acceptable product (visual quality and aroma), while the application of marjoram EO was able to preserve the visual quality of fresh-cut lettuce and at the same time resulted in a pleasant aroma. The application of EO+AA and Chitosan+AA increased total phenolics and antioxidant levels of fresh-cut lettuce on the fourth and sixth day of storage. The EO and EO+AA increased damage index (hydrogen peroxide and lipid peroxidation) of fresh-cut lettuce, while at the same time these treatments decreased the activity of enzymes related with plant tissue browning (i.e., peroxidase activity and polyphenol oxidase). Chitosan decreased total valuable counts and yeasts and molds counts on the sixth day of storage, while EO, AA, EO+Chitosan, and Chitosan+AA decreased yeasts and molds after four days of application. The findings of the present work indicating that the combination of marjoram EO, AA, and chitosan could be considered further as alternative means for fresh-cut produce preservation.

## 1. Introduction

Over the last few years, the market of fresh-cut produce has been growing dramatically as the increased consumer demand for fresh, safe, healthy (high nutritional value), and convenient food [1]. Vegetables (including leafy, roots, and other plants’ part) that are intended to be marketed as “fresh-cut” are subjected to washing, peeling, slicing, and shredding followed by packing and sealing in polymeric films [2]. Lettuce is quite popular and consumed widely due to its crispness, pleasant aroma, and high levels of phytonutrients, such as phenolic components and vitamins (C, K, and folate). However, lettuce is a very perishable vegetable, and when processed and/or stored at less favored conditions (i.e., high temperature, low humidity, and improper packaging material) it is susceptible to physiological disorders, enzymatic browning, loss of nutritional value, microbial contamination, and spoilage [1,3,4]. Several studies assessed the microbial quality of ready-to eat vegetables and salads revealing a broad range of microorganisms (bacteria, fungi, and viruses) that can possible adversely affect product quality (spoilage) and/or threaten human health (foodborne pathogens) [5,6,7,8].

The washing procedure during minimal processing is performed using chlorinated water which contains 0.05–0.2 g L^−1^ sodium hypochloride (NaOCl), and the applied dose (concentration and time of application) depends on the product (fresh produce) [9]. The main aim of washing is the reduction of the microbial load of vegetables [2]. However, as it has been previously reported, chlorine can cause the production of undesirable and harmful by-products like trihalomethanes, haloketones, chlorophorm, and halloacetic acids with adverse effects in human health as well as the environment [2,10]. This has led to increased concern and demand for natural alternatives to replace chemicals used in the food industry and meet consumers’ needs. These alternatives might include essential oils (EOs) from medicinal and aromatic plants, organic acids (ascorbic, oxalic, and lactic acid), peptides, reducing agents (ascorbic acid and cysteine), and more [2,11,12,13,14,15,16,17].

Ascorbic acid (AA) is being extensively used in the food industry mainly as a reducing agent in order to prevent undesirable reactions such as enzymatic browning. As with other organic acids (i.e., citric and oxalic acid), AA has been used during processing in order to regulate the microbial load of fresh commodities due to its antioxidant activity as well as its ability to lower the pH of washing water [2,16,18,19]. The application of AA on fresh-cut lettuce has been previously reported with promising results [18,19,20,21]. For instance, Altunkaya and Gökmen [18] reported that AA maintained high phenolic content of fresh-cut lettuce indicating lower phenol oxidation levels due to enzymatic activity and thus lower browning on cut surfaces. In another study, the bright green color of lettuce was maintained with the application of AA [20].

Essential oils are plants’ secondary metabolites that have been used for prolonging food shelf life and improving organoleptic attributes (aroma and taste) of food as many of them are characterized as Generally Recognized as Safe (GRAS) [22]. They possess antioxidant and amicrobial activity among others and can be used in low levels that are safe for consumption [23]. The application of EOs on fresh produce has been previously mentioned and several research studies have tested the impacts of EO application on fresh-cut lettuce [4,13,24,25]. The application of EOs on minimally processed vegetables such as lamb’s lettuce, shredded carrot, sliced cucumber, and broccoli has been previously reported with encouraging results [15,26,27]. For example, the use of marjoram EO and hydrosol when combined with AA improved the quality of shredded carrot stored at 4 °C for nine days [15].

Chitosan is a naturally occurring polymer delivered from crab shells, and it has been employed to stabilize ingredients in tablets in medicines. Chitosan was the first basic compound for plant protection that was approved by the European Union (Reg. EU 2014/563) due to its low toxicity and when applied to plants its effectiveness results from three mechanisms (film formation, antimicrobial activity, and elicitation of host defense) [28]. It has been previously shown that edible chitosan coatings alone or incorporated with EOs presented enhanced antimicrobial activity against foodborne pathogens including *Pseudomonas aeruginosa*, *Staphylococcus aureus*, *Bacillus cereus*, *Serratia marcescens*, *Escherichia coli*, *Enterococcus faecalis*, and *Salmonella enteritidis* [29]. Chitosan has been proven to act as an elicitor of plant defense mechanisms when applied to plant tissues and this forms a protection against pre- and postharvest microbial contamination and spoilage of strawberry fruit as reported by Landi et al. [30]. Chitosan and commercial chitosan formulations have been previously applied to fresh produce (dipping and spraying) with encouraging results [31,32]. However, little is known about the chitosan application on fresh-cut produce.

The aim of the present study was to evaluate the effectiveness of marjoram EO, AA, chitosan, and different combinations of them in the preservation of quality-related attributes of fresh-cut lettuce stored under commercial temperature (7 °C).

## 2. Materials and Methods

### 2.1. Plant Material and EO Extraction

Fresh lettuce (*Lactuca sativa* L.) was obtained from a local market in Limassol, Cyprus, and samples were selected to be uniform in appearance and the absence of physical defects or injury and then stored at 4 °C and 90% relative humidity (RH) until use (within 24 h).

Marjoram plants (*Origanum majorana* L.) grown in soil for two years were harvested from the experimental farm of Cyprus University of Technology. Plant tissue was air-dried (in oven at 42 °C) and chopped, and hydrodistillation was performed using Clevenger apparatus for 3 h for the extraction of the EO. The obtained EO was stored at −20 °C until use in amber glass vials. Essential oil composition was determined as described previously [15], and the main components were terpinen-4-ol, γ-terpinene, *trans*-sabinene hydrate, and α-terpinene.

Unless otherwise stated, all chemical reagents were analytical grade and were purchased from Sigma-Aldrich (A.J. Vouros Ltd., Nicosia, Cyprus).

### 2.2. Preliminary Screening

After the removal of damaged exterior leaves, fresh lettuce leaves were chopped into pieces (5 × 10 cm) and washed with tap water. Then, 70 g of fresh-cut leaves was dipped for 1 min (based on preliminary tests following evaluation for the dipping time of 1, 5, and 10 min during a 4-day storage, as presented in Figure S1) into 0.5 L of treatment solution. The following 14 dipping solutions were studied: (1) distilled water (control), (2) marjoram EO (1:1000, 1:1500, 1:2000, and 1:2500 *v/v*), (3) chitosan (Chito Plant; ChiPro GmbH, Bremen, Germany) (0.1, 0.125, 0.25, 0.5 and 1% *w/v*), and (4) ascorbic acid (Scharlau, Spain) (0.25, 0.5, 1, and 2% *w/v*). Afterwards, leaf pieces were drained, and ~15 g was placed in a polypropylene plastic container (1 L) and enclosed into a polyethylene terephthalate plastic tray and stored at 7 °C and 90% RH until the day of sampling (days 0, 2, 4, and 6). Three biological replicates for each treatment/concentration were sampled and plant tissue was stored at −20 °C until analysis. For preliminary screening, aroma and marketability were assessed by seven untrained panelists, while weight loss and total polyphenol content were determined and used for choosing the adequate dipping solutions for further assessment.

Lettuce weight loss was monitored every second day during storage at 7 °C for 6 d (days 0, 2, 4, and 6) and results were presented as percentage of total weight loss. After storage for 6 d overall aroma and chroma/color (visual quality) were scored. Evaluation of aroma was assessed with a 5-point hedonic scale (0.5 interval) where 5 = not acceptable; 3 = not lettuce but acceptable; 1 = lettuce. Visual quality was evaluated with a 5-point hedonic scale (0.5 interval) where 5 = severe browning; 3 = light discoloration; 1 = green. Total phenols content of methanolic extracts was determined at 755 nm using a spectrophotometer (Multiskan GO, Thermo Fisher Scientific Oy, Vantaa, Finland), as previously described by Marinou et al. [33] and results were expressed as g of gallic acid equivalents (GAE) per kg of fresh weight (g kg^−1^ GAE).

### 2.3. Main Study for the Determination of Quality and Antioxidant Activity

Following preliminary screening, lettuce leaves were selected, prepared, and processed as above. Then, ~70 g of fresh-cut lettuce was dipped for 1 min into 0.5 L of treatment solution selected from the preliminary screening. The following eight dipping solutions/combinations were further assessed: (1) distilled water (control), (2) marjoram EO (1:1500 *v/v*), (3) chitosan (0.1% *w/v*), (4) ascorbic acid (1% *w/v*), (5) marjoram EO (1:1500) + chitosan (0.1%), (6) marjoram EO (1:1500) + ascorbic acid (1%), (7) chitosan (0.1%) + ascorbic acid (1%), and (8) chlorine (0.2 mL L^−1^). Afterwards, lettuce pieces were drained, and ~70 g of lettuce pieces was placed into a polypropylene plastic tray and stored at 7 °C and 90% RH until the day of sampling (days 0, 2, 4, and 6). Three biological replicates per treatment/day were sampled, while appropriate amount of tissue was stored at −20 °C until analysis.

#### 2.3.1. Weight Loss and Color

Weight loss was determined as mentioned above and results were expressed as percentage of total weight loss. Minimally processed lettuce’s color was evaluated using a colorimeter (Chroma meter CR400 Konica Minolta, Japan) and L*, a*, b* values were recorded. Chroma value (C), hue (h), and whiteness index (WI) were calculated as C = (a*^2^ + b*^2^)^1/2^, h = tan^−1^(b*/a*), and WI = 100 − [(100 − L*)^2^ + a*^2^ + b*^2^]^1/2^ [34,35]. Color index (CI) was calculated as CI = (a* × 1000)/(L* × b*) [36]. Browning index (BI) was calculated as BI = 100 × (X − 0.31)/0.17, where X = (a* + 1.75 × L*)/(5.645 × L* + a* − 3.012 × b*) [37].

#### 2.3.2. Respiration Rate and Ethylene Emission

Respiration of the fresh-cut lettuce was evaluated by measuring the CO_2_ concentration as previously described [15]. Briefly, samples were enclosed in a polypropylene plastic container (1 L) at room temperature for 1 h, container’s air was withdrawing by a dual gas analyzer (GCS 250 Analyzer, International Control Analyser Ltd., Kent, UK) for 40 s and respiration rate was expressed as mg of CO_2_ per kg of fresh weight per h (mg kg^−1^ h^−1^ CO_2_) according to the volume and weight of the processed lettuce. Ethylene flow rate was estimated by measuring the ethylene concentration of the packages as previously mentioned by Chrysargyris et al. [17]. Briefly, air was withdrawn from sample containers by an ethylene analyzer (ICA 56 Analyser, International Control Analyser Ltd., Kent, UK) for 20 s through a hole on the lid recording the ppm of ethylene produced, and ethylene emission was computed as μg of ethylene per kg of fresh weight per h (μg kg^−1^ h^−1^ ethylene).

#### 2.3.3. Leaf Chlorophyll and Carotenoid Content

For leaf pigment extraction and determination, the procedure was performed using dimethyl sulfoxide (Merck, Darmstadt, Germany) as reported by Wellburn [38] and results were expresses as g chlorophyll (or carotenoids) per kg of fresh weight (g kg^−1^ chl or car).

#### 2.3.4. Total Soluble Solids, Total Acidity, Ascorbic Acid

Lettuce tissue (three biological replicates/treatment/day; 1 g) was grinded/pressed to extract the juice with a domestic blender. Total soluble solids (TSS) content was determined using a digital portable refractometer (Atago, Tokyo, Japan) and results were expressed in percentage (%). Lettuce titratable acidity (TA) was assessed by titration with 0.1 N NaOH as previously reported [39] and results are expressed as percentage malic acid (% TA). Sweetness as ratio of TSS over TA (TSS/TA) was also evaluated.

The quantification of AA content was assessed by titration 2,6-dichlorophenol-indophenol and results were expressed as g of AA per kg of fresh weight (g kg^−1^ AA) [15].

#### 2.3.5. Total Phenols Content and Antioxidant Activity

Total phenols content was determined as previously mentioned (Section 2.2) and results are expressed as g of GAE per kg of fresh weight (g kg^−1^ GAE). The 2,2-diphenyl-1-picrylhydrazyl (DPPH) and 2,2′-azino-bis(3-ethylbenzothiazoline-6-sulphonic acid) (ABTS) assays were used for the determination of antioxidant activity of lettuce methanolic extracts. The antioxidant capacity using the DPPH method was determined at 517 nm as previously described by Wojdylo et al. [40], using a spectrophotometer (Multiskan GO, Thermo Fisher Scientific Oy, Finland). The reduction of the ABTS radical was assessed at 734 nm as previously described by Wojdylo et al. [40], using the same spectrophotometer as above. Results expressed as g trolox per kg of fresh weight (g kg^−1^ trolox).

#### 2.3.6. Hydrogen Peroxide and Lipid Peroxidation

The concentration of hydrogen peroxide (H_2_O_2_) was quantified based to the method described previously by Loreto and Velikova [41] at 390 nm and results were expressed as nmol of H_2_O_2_ per kg of fresh weight (nmol kg^−1^ H_2_O_2_). Lipid peroxidation of minimally processed lettuce was evaluated using the 2-thiobarbituric acid reactive substances method measuring the absorbance at 532 and 600 nm as previously described [42], using a spectrophotometer (Multiskan GO, Thermo Fisher Scientific Oy, Finland). Results were expressed as nmol of malondialdeyde (MDA) per kg of fresh weight (nmol kg^−1^ MDA).

#### 2.3.7. Activity of Antioxidant and Browning Enzymes

For the preparation of crude extracts, fresh leaf samples were homogenized with ice cold 50 mM potassium phosphate buffer (pH 7.0), containing 1 mM phenylmethylsulfonyl fluoride (Merck, Darmstadt, Germany), 1 mM ethylenediaminetetraacetic acid, 1% *w/v* polyvinylpolypyrrolidone, and 0.05% polyethylene glycol tert-octylphenyl ether (Triton X-100). The homogenate was transferred to centrifuge tubes and was centrifuged at 16,000× *g* for 15 min, at 4 °C [42]. The supernatant was transferred to new tubes and an aliquot was used to determine the protein content according to the Bradford method [43], using bovine serum albumin as standard. The extract was then used for the measurement of enzyme activity, using a spectrophotometer (Multiskan GO, Thermo Fisher Scientific Oy, Finland).

Catalase activity (CAT, EC 1.11.1.6) was measured according to Jiang and Zhang [44], following the consumption of H_2_O_2_ at 240 nm, for 3 min. Results were expressed as CAT units per milligram of protein. One unit of enzyme decomposes 1 μmol of H_2_O_2_ per min (coefficient of extinction 39.4 mM cm^−1^).

The photochemical method was used to determine the activity of superoxide dismutase (SOD, EC 1.15.1.1), as described by Chrysargyris et al. [42]. SOD activity was calculated from the absorbance of the reaction mixture at 560 nm, as compared to the control. Results were expressed as SOD units per mg of protein, while one unit of SOD was defined as the enzyme inhibiting the NBT reduction by 50%.

Peroxidase activity (POD, EC 1.11.1.7) was assayed using pyrogallol as a substrate [45]. The increase in absorbance at 430 nm was monitored for 3 min. One POD unit was defined as the amount of enzyme that decomposes 1 μmol of H_2_O_2_ per min. Results were expressed as units of POD per milligram of protein (coefficient of extinction of 2.47 mM cm^−1^).

For polyphenol oxidase (PPO, EC 1.10.3.1) activity, the enzyme extract was mixed with 2.4 mL of 20 mM catechol in 50 mM sodium phosphate buffer (pH 7.0). Absorbance was measured at 420 nm, and one unit of enzyme activity was defined as an increase in absorbance at 0.01 min^−1^. Results were expressed as units of enzyme per mg of protein [46].

#### 2.3.8. Microbiological Analysis

Total viable count (TVC) as well as yeast and molds were determined using Plate count agar (PCA, Merck, Darmstadt, Germany) and Rose Bengal Chloramphenicol Agar (Liofilchem s.r.l, Italy), respectively, as previously described by Chen et al. [47]. Results were expressed as log CFU per g of fresh weight (log CFU g^−1^).

#### 2.3.9. Sensory Evaluation

Sensory evaluation (aroma and visual quality) was performed on each sampling day, as described above (Section 2.2). Briefly, aroma was evaluated with a 5-point hedonic scale (0.5 interval) where 5 = not acceptable; 3 = not lettuce but acceptable; 1 = lettuce. Visual quality was evaluated with a 5-point hedonic scale (0.5 interval) where 5 = severe browning; 3 = light discoloration; 1 = green.

### 2.4. Statistical Analysis

The experiment was performed according to the randomized complete block design. The analysis of data was accomplished by comparing means with one-way analysis of variance (ANOVA) using IBM SPSS version 25, and Duncan’s multiple range test was performed for *p* = 0.05. Three (n = 3) biological replicates were used, and values refereed to mean ±SE. Microbiological analysis was done with duplicate plates for each of the three replicates. Results are expressed on a fresh weight basis.

## 3. Results

### 3.1. Preliminary Screening

**Figure 1:**
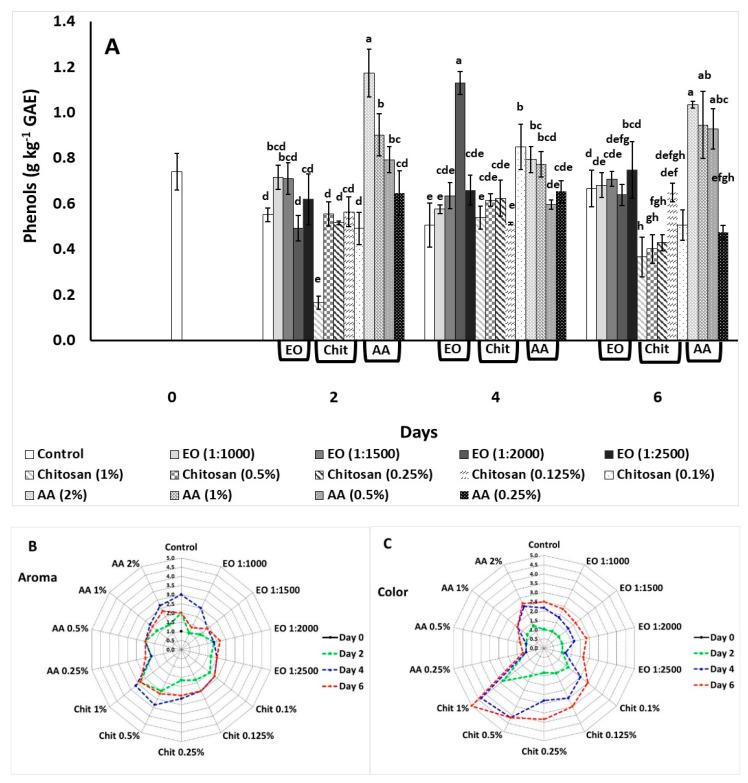
Screening of marjoram essential oil (EO), chitosan (Chit), and ascorbic acid (AA) on fresh-cut lettuce (**A**) total phenols (g kg^−1^ GAE), (**B**) aroma (scale range from 1—lettuce like to 5—not lettuce, not acceptable), and (**C**) color/visual quality (scale: 1—green; 3—light discoloration; 5—severe browning) after 6 days of storage at 7 °C and 90% RH. On the columns, significant differences (*p* < 0.05) among treatments are indicated by different Latin letters for different days. Values of phenolics represent means (±SE) of measurements on three biological replicates per treatment. Aroma and color evaluation were assessed by 7 untrained panelists. GAE, gallic acid equivalents.

The effect of marjoram EO, chitosan, and AA application on fresh-cut lettuce during the preliminary screening is shown in Figure 1 and Figure S2. During storage, EO application (1:1500) reduced and AA of 0.5% accelerated weight loss after 2 days compared with the control treatment (Figure S2). Following 6 days of storage, EO application (1:1500) and chitosan of 0.125% reveled lower weight losses while all the other applications had same weight losses as the control treatment.

Total phenols content was increased with the use of AA 2%, 1%, and 0.5% and EO from 1:1000 to 1:2500 compared to the control treatment after 2 days of storage, while chitosan 1% resulted to lower phenolics on the same day (Figure 1A). During the fourth day of storage an increase of phenols was found with the application of EO 1:2000, AA 2%, 1% and 0.5%, and chitosan 0.1% compared to the control treatment. Phenolic content was higher after 6 days with the application of AA 2%, 1%, and 0.5%, while the application of AA 0.25% decreased the phenolic content compared to the control treatment. On the same day, decreased phenolics were reported with the application of chitosan 1%, 0.5%, and 0.25%, while no significant difference was found between the application of EO and the control treatment.

The effects of the applied preservative means on the visual quality and aroma of fresh-cut lettuce are illustrated in Figure 1 and Figure S3. EO and AA (2% and 1%, respectively) applications maintained the green color of fresh-cut lettuce throughout storage (up to 6 days). The application of chitosan on higher concentrations (1% and 0.5%) resulted to higher scores on the fourth and sixth day of storage compared to other treatments (Figure 1C). Note that chitosan 1% scored 4 out of a 5 scale reflecting the incidence of browning on fresh-cut lettuce from the second day of storage. Moreover, the application of chitosan 0.5 and 1% after four and six days of storage resulted in a product scored with 3 out of a 5 scale, which indicated an acceptable aroma but not lettuce like (Figure 1B). On the other hand, the application of AA and EO 1:1000 and 1:1500 revealed a lettuce like (“freshness”) aroma scoring 1 and 2 out of a 5 scale.

### 3.2. Main Experiment

#### 3.2.1. Weight Loss, Respiration Rate and Ethylene Emission

**Figure 2:**
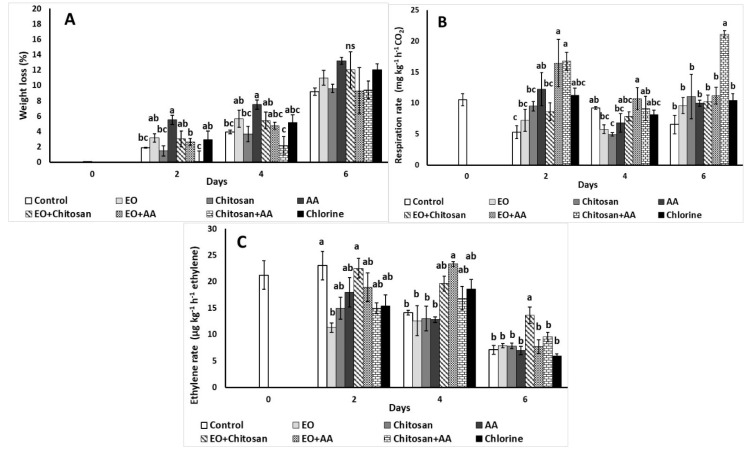
Effect of marjoram essential oil (EO), chitosan, and ascorbic acid (AA) on fresh-cut lettuce (**A**) weight loss (%); (**B**) respiration (mg kg^−1^ h^−1^ CO_2_); and (**C**) ethylene (μg kg^−1^ h^−1^ ethylene) rate after treatment and 6 days of storage at 7 °C and 90% RH. On the columns, significant differences (*p* < 0.05) among treatments are indicated by different Latin letters; ns indicates no significant differences for different days. Values represent means (±SE) of measurements made on three biological replicates per treatment.

The application of AA increased (up to 5.53%) weight loss of fresh-cut lettuce on the second day of storage compared to control, chitosan, Chitosan+AA, EO+AA, and chlorine (Figure 2A). A similar trend was observed after 4 days of storage but not at the sixth day, where all treatments revealed similar weight losses.

Respiration rate was increased on the second day with the application of EO+AA, EO+Chitosan, and AA in comparison to the control treatment (Figure 2B). Chitosan application resulted in lower respiration rate on the fourth day in comparison to the control treatment and the combinations of EO+AA and Chitosan+AA. Following that an increased respiration rate was also observed on the sixth day with the combination of Chitosan+AA that revealed the highest value compared to all treatments.

Ethylene emission as affected by the application of the natural products is illustrated in Figure 2C. The application of EO resulted to lower ethylene production compared to the control and EO+Chitosan treatments on the second day of storage. On the fourth day, increased ethylene emission was reported with the combination of EO+AA as to the control treatment, EO and Chitosan+AA, while on the sixth day, the application of EO+Chitosan resulted to higher ethylene production compared to all treatments.

#### 3.2.2. Color Indices

**Figure 3:**
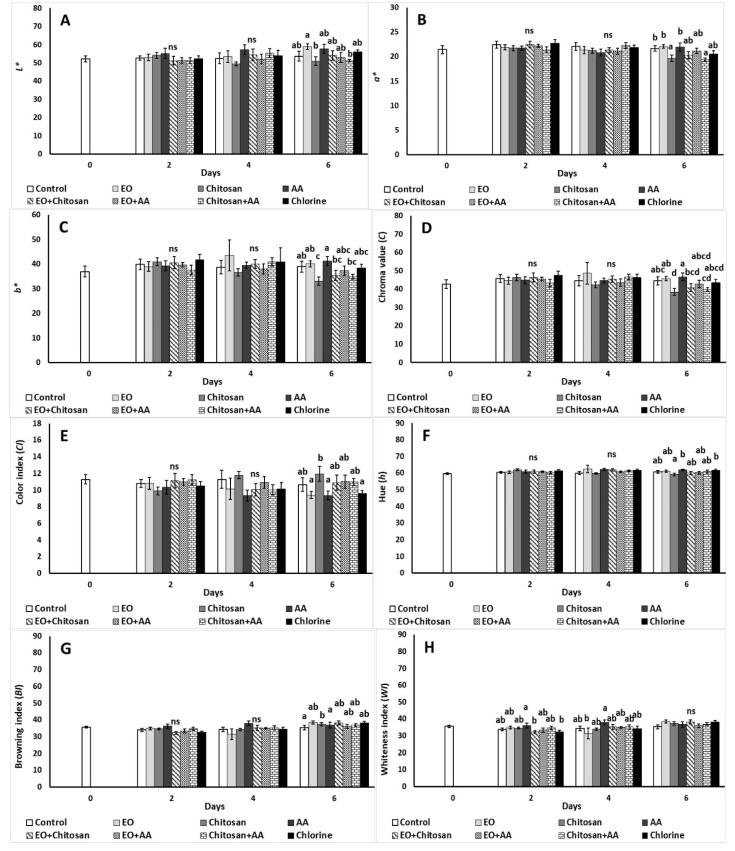
Effect of marjoram essential oil (EO), chitosan, and ascorbic acid (AA) on fresh-cut lettuce on (**A**) L* value, (**B**) a* (greenness) (positive values), (**C**) b* (yellowness), (**D**) chroma, (**E**) color index (positive values), (**F**) hue (positive values), (**G**) browning index, and (**H**) whiteness index after treatment and 6 days of storage at 7 °C and 90% RH. On the columns, significant differences (*p* < 0.05) among treatments are indicated by different Latin letters; ns indicates no significant differences for different days. Values represent means (±SE) of measurements made on three biological replicates per treatment.

The impact of marjoram EO, chitosan, and AA application and their combinations on fresh-cut lettuce color is illustrated in Figure 3. The utilization of EO increased L* value compared to chitosan and Chitosan+AA after 6 days of storage (Figure 3A). Lower a* values (positive values are shown in Figure 3B) were reported on the sixth day of storage with the application of chitosan and Chitosan+AA. Similarly, decreased b* values (positive values are shown in Figure 3C) were observed with the utilization of chitosan, EO+Chitosan and Chitosan+AA. Chroma value decreased with the application of chitosan, EO+Chitosan, and Chitosan+AA on the sixth day, while increased CI value (positive values are shown in Figure 3D,E) was reported with the application of chitosan compared to EO, AA, and chlorine on the same day. Chitosan application decreased Hue (59.16) compared to the application of AA and chlorine (61.92 and 61.80, respectively) on the sixth day (positive values are shown in Figure 3F). Browning index decreased on the 6th day with the application of chitosan compared to the control treatment, while WI increased by using AA compared to EO+Chitosan and chlorine on the second day (Figure 3G,H). On the fourth day of storage, AA was found to have an increased WI in comparison to EO application.

#### 3.2.3. Chlorophyll Content (Chl a, Chl b, Total Chl)

**Figure 4:**
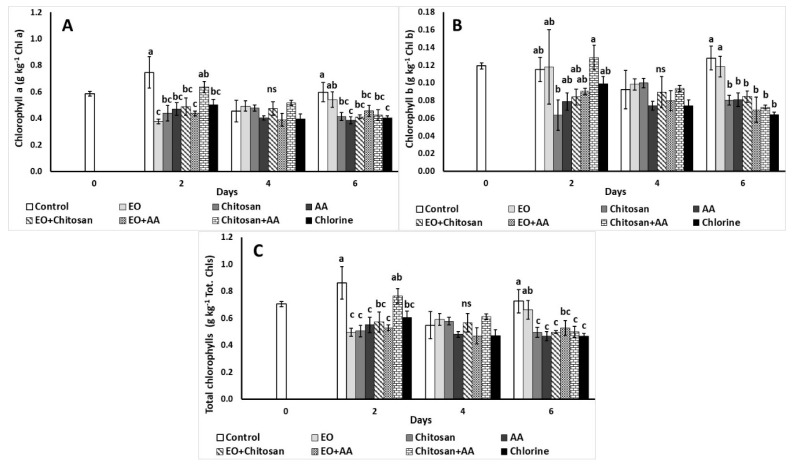
Effect of marjoram essential oil (EO), chitosan and ascorbic acid (AA) on fresh-cut lettuce (**A**) chlorophyll a (g kg^−1^), (**B**) chlorophyll b (g kg^−1^), and (**C**) total chlorophyll (g kg^−1^) content after treatment and 6 days of storage at 7 °C and 90% RH. On the columns, significant differences (*p* < 0.05) among treatments are indicated by different Latin letters; ns indicates no significant differences for different days. Values represent means (±SE) of measurements made on three biological replicates per treatment.

The effects of postharvest treatments applied on fresh-cut lettuce chlorophyll content are illustrated in Figure 4. All applied treatments except EO decreased chlorophyll content (Chl a, Chl b, Tot Chl) on the sixth day of storage. Chitosan application decreased Chl b levels compared to Chitosan+AA on the second day, while the same trend on the same day was observed with Chl a content (Figure 4A,B). Additionally, Tot Chl levels decreased with the utilization of Chitosan+AA during the second day of storage (Figure 4C).

#### 3.2.4. Total Soluble Solids, Total Acidity, and Sweetness

**Figure 5:**
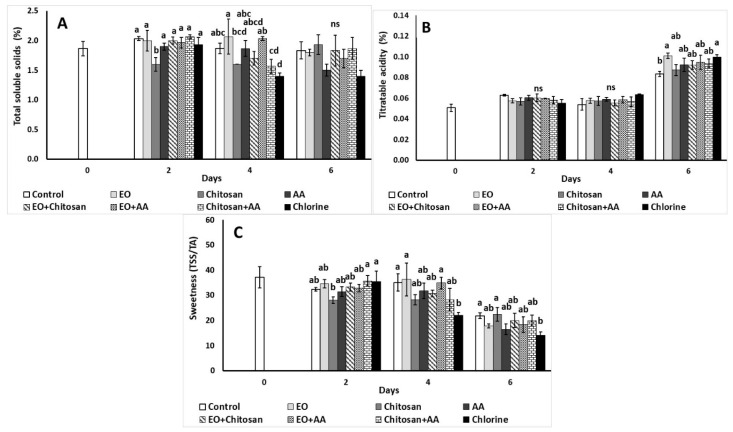
Effect of marjoram essential oil (EO), chitosan and ascorbic acid (AA) on fresh-cut lettuce (**A**) total soluble solids (TSS; %), (**B**) total acidity (TA; %), and (**C**) sweetness (TSA/TA) after treatment and 6 days storage at 7 °C and 90% RH. On the columns, significant differences (*p* < 0.05) among treatments are indicated by different Latin letters; ns indicates no significant differences for different days. Values represent means (±SE) of measurements made on three biological replicates per treatment.

Total soluble solids of fresh-cut lettuce were decreased with the application of chitosan on the second day of storage, while EO application increased TSS compared to chitosan, Chitosan+AA, and chlorine on the fourth day (Figure 5A). At the end of storage (sixth day), an increase of TA was found with the use of EO and chlorine in comparison to the control at day 6 (Figure 5B). As shown in Figure 5C, sweetness increased with the combination of Chitosan+AA and chlorine as to chitosan on the second day of storage, while the chlorine decreased sweetness of fresh-cut lettuce on the fourth day when compared to the control treatment, EO, and EO+AA. Similarly, on the sixth day of storage chlorine caused decreased sweetness opposed to the control treatment and chitosan.

#### 3.2.5. Total Phenols, Antioxidants, Ascorbic Acid, and Total Carotenoid Content

**Figure 6:**
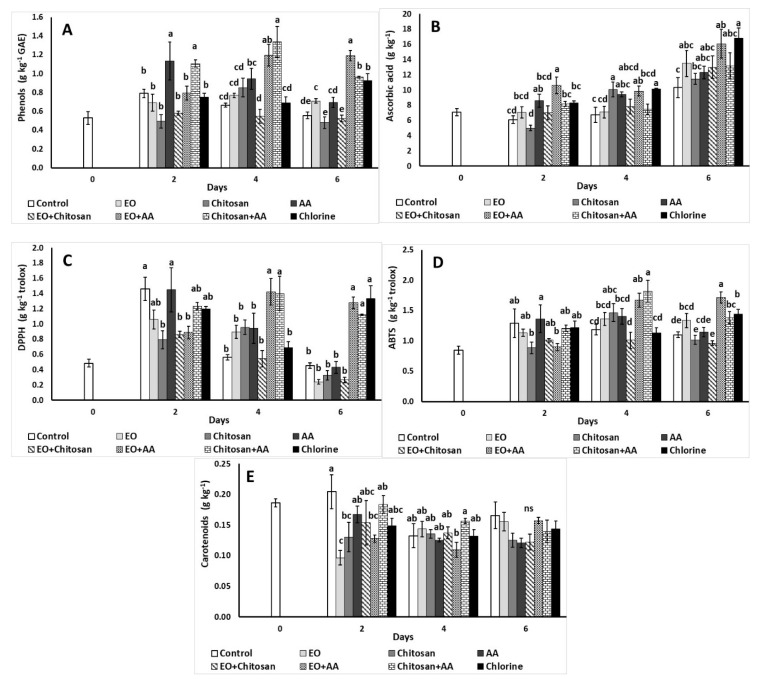
Effect of marjoram essential oil (EO), chitosan and ascorbic acid (AA) on fresh-cut lettuce (**A**) total phenolics (g kg^−1^ GAE), (**B**) ascorbic acid (g kg^−1^), (**C**) antioxidant activity (DPPH; g kg^−1^ trolox), (**D**) antioxidant activity (ABTS; g kg^−1^ trolox), and (**E**) carotenoids (g kg^−1^) content after treatment and 6 days of storage at 7 °C and 90% RH. On the columns, significant differences (*p* < 0.05) among treatments are indicated by different Latin letters; ns indicates no significant differences for different days. Values represent means (±SE) of measurements made on three biological replicates per treatment.

Total phenols content was increased with the application of AA and Chitosan+AA on the second day as presented in Figure 6A. On the fourth day, EO+AA and Chitosan+AA increased phenolics and similarly EO+AA, Chitosan+AA, and chlorine resulted to higher phenolic content (1.19, 0.96, and 0.92 g kg^−1^ GAE, respectively) at the end of storage.

Ascorbic acid content was increased with the application of AA and EO+AA on the second day, while chitosan lowered ascorbic acid of fresh-cut lettuce on the same day (Figure 6B). Moreover, increased ascorbic acid content was revealed with the application of chitosan, chlorine, EO+AA, and AA higher values on the fourth day, while EO+AA and chlorine increased ascorbic acid during the last day of storage.

Total carotenoid content decreased with the use of EO, chitosan and EO+AA on the second day of storage, while the combination of Chitosan+AA, increased carotenoids compared to EO+AA during the fourth day of storage (Figure 6E).

The antioxidant capacity of fresh-cut lettuce decreased with the application of chitosan, EO+Chitosan and EO+AA (DPPH: 0.79, 0.86, and 0.89 g kg^−1^ trolox, respectively) compared to the control treatment and AA (1.46 and 1.45 g kg^−1^ trolox, respectively) on the second day (Figure 6C), while EO+AA and Chitosan+AA resulted to higher DPPH antioxidant levels on the fourth day. Similarly, the application of EO+AA, Chitosan+AA, and chlorine increased DPPH antioxidants during the sixth day. The AA application increased lettuce antioxidants on the second day compared to the application of chitosan and EO+AA (Figure 6D), while the combinations of EO+AA and Chitosan+AA resulted to higher ABTS compared to control, EO, EO+Chitosan, and chlorine during the fourth day. However, Chitosan and EO+Chitosan lowered lettuce ABTS antioxidants during the last day of storage compared to EO, EO+AA, Chitosan+AA, and chlorine.

#### 3.2.6. Hydrogen Peroxide and Lipid Peroxidation

**Figure 7:**
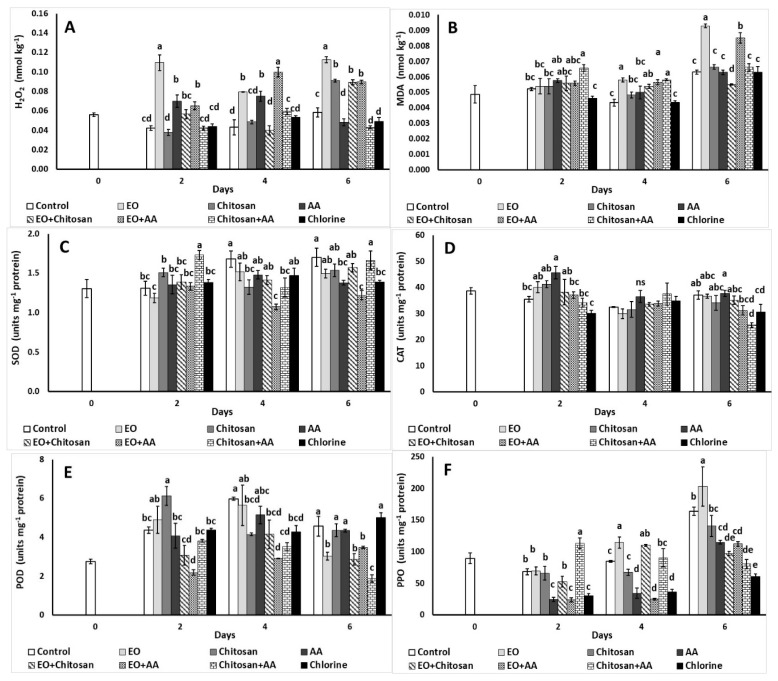
Effect of marjoram essential oil (EO), chitosan, and ascorbic acid (AA) on fresh-cut lettuce on (**A**) H_2_O_2_ production (nmol kg^−1^), (**B**) lipid peroxidation (nmol kg^−1^) and antioxidant enzymes activity of (**C**) superoxide dismutase (SOD; units mg^−1^ protein), (**D**) catalase (CAT; units mg^−1^ protein), (**E**) peroxidase (POD; units mg^−1^ protein), and (**F**) polyphenol oxidase (PPO; units mg^−1^ protein) after treatment and 6 days of storage at 7 °C and 90% RH. On the columns, significant differences (*p* < 0.05) among treatments are indicated by different Latin letters; ns indicates no significant differences for different days. Values represent means (±SE) of measurements made on three biological replicates per treatment.

The effect of marjoram EO, chitosan, and AA application on hydrogen peroxide and lipid peroxidation is illustrated in Figure 7. Increased H_2_O_2_ levels were reported in case of marjoram EO, AA, and EO+AA applications on the second and fourth day of storage. During the last day of storage, the application of AA, Chitosan+AA, and chlorine led to lower H_2_O_2_ levels, while EO application showed a higher value (Figure 7A).

Lipid peroxidation indicated by MDA was increased on the second day with Chitosan+AA compared to the control treatment and chlorine (Figure 7B), while increased MDA production was also reported with EO, EO+AA, and Chitosan+AA application on the fourth day. During the last day of storage EO and EO+AA led to increased MDA production, while EO+Chitosan resulted to lower MDA levels.

#### 3.2.7. Antioxidant and Browning Enzymes

The application of Chitosan+AA increased SOD activity of lettuce compared to other treatments on the second day, while on the fourth day chitosan and EO+AA decreased SOD activity (1.32 and 1.07 units mg^−1^ protein, respectively) compared to control (1.68 units mg^−1^ protein) (Figure 7C). On the last day of storage, SOD activity was decreased with AA, EO+AA, and chlorine compared to control. AA increased CAT activity on the second day of storage compared to control, while Chitosan+AA and chlorine decreased CAT activity on the last day in comparison to the control (Figure 7D). POD activity increased on the second day with the application of chitosan, while EO+AA decreased its activity on the same day compared to control (Figure 7E). Chitosan, EO+Chitosan, EO+AA, Chitosan+AA, and chlorine decreased POD activity on the fourth day of storage, while EO, EO+Chitosan, EO+AA, and Chitosan+AA also decreased its activity on the last day of storage. Chitosan+AA application led to increased PPO activity on the second day of storage (113.07 units mg^−1^ protein), while deceased enzyme’s activity was found on the last day of storage compared to control (Figure 7F). Moreover, the application of AA, EO+AA, and chlorine decreased PPO activity after the second day and throughout storage, while EO, EO+Chitosan application increased PPO activity after the fourth day of storage.

#### 3.2.8. Microbial Load

**Figure 8:**
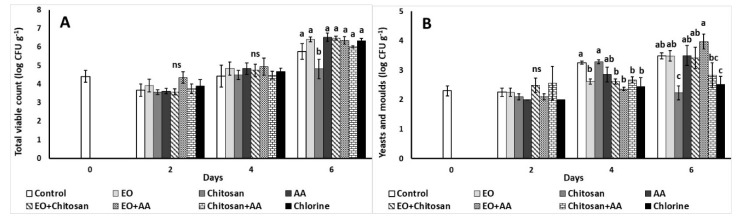
Effect of marjoram essential oil (EO), chitosan and ascorbic acid (AA) on shredded carrots on (**A**) total viable count (TVC; log CFU g^−1^) and (**B**) yeast and molds (log CFU g^−1^) after 6 days of storage at 7 °C and 90% RH. On the columns, significant differences (*p* < 0.05) among treatments are indicated by different Latin letters; ns indicates no significant differences for different days. Values represent means (±SE) of measurements made on three biological replicates per treatment.

Figure 8 shows the impacts of the preservative means applied on fresh-cut lettuce microbial quality. Chitosan application resulted to decreased total viable counts (4.58 log CFU g^−1^) during the last day of storage (Figure 8A). The application of EO, EO+Chitosan, EO+AA, Chitosan+AA and Chlorine resulted to decreased yeast and molds (2.62, 2.62, 2.67, and 2.45 log CFU g^−1^, respectively) on the fourth day of storage compared with the control treatment, while chitosan and chlorine decreased yeast and molds compared to control, EO, AA, EO+Chitosan, and EO+AA on the sixth day (Figure 8B).

#### 3.2.9. Sensory Characteristics

**Figure 9:**
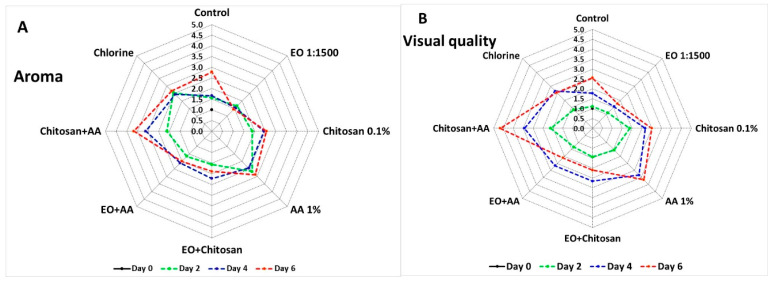
Effects of marjoram essential oil (EO), chitosan, ascorbic acid (AA) alone, and their combinations on fresh-cut lettuce (**A**) aroma (scale range from 1—lettuce like to 5—not lettuce, not acceptable) and (**B**) color/visual quality (scale: 1—green; 3—light discoloration; 5—severe browning) after 6 days of storage at 7 °C and 90% RH. Values represent means of evaluations made by 7 untrained panelists.

Figure 9 presents the impacts of the applied treatments on the aroma and visual quality of fresh-cut lettuce. The combination of Chitosan+AA scored higher values (4 out of a 5 scale) than all the applied treatments during the fourth and sixth days of storage, indicating a non-acceptable product (Figure 9A). Furthermore, the application of EO scored lower values during all days suggesting an acceptable product with a pleasant/acceptable aroma (1–2 out of a 5 scale). Moreover, the visual quality of fresh-cut lettuce decreased with the application of Chitosan+AA and chitosan, as these treatments were scored higher with high values (3–4 out of a 5 scale) during all days of storage (Figure 9 and Figure S4). Increased values were also revealed with the use of AA during the fourth and sixth day (3 out of a 5 scale), while EO application preserved the visual quality of fresh-cut lettuce during all days of storage (scores 1–2 out of a 5 scale) (Figure 9B).

## 4. Discussion

Fresh vegetables are one of the main components in a healthy and balanced diet, as they are high in dietary fiber and phytonutrients with low fat content and calories. However, when exposed to adverse handling and storage conditions, vegetables (especially minimally processed) tend to have short shelf life due to deterioration of their quality attributes. The use of natural products on fresh commodities and minimally processed fruit and vegetables has been previously studied by researchers and the outcomes are promising [14,15,17,30,32,48,49,50], but most importantly are well appreciated by the consumers. In the present work, the effects of the application of marjoram EO, chitosan, AA, and their combinations were assessed regarding the quality attributes of fresh-cut lettuce, indicating an alternative preservation postharvest management for quality and safe stored products.

Chitosan application negatively impacted the aroma and visual quality of fresh-cut lettuce. A non-acceptable aroma and less attractive color was found with the use of chitosan and Chitosan+AA. On the other hand, EO application improved visual quality and acceptable (pleasant) aroma. Similarly, the use of AA maintained the visual quality of fresh-cut lettuce. The application of chitosan-based melatonin bilayer coating on ready-to eat cucumber and broccoli was assessed by Zhao et al. [27], and their results supported the benefits observed by combining postharvest preservation means, showing that the applied combinations of melatonin and chitosan maintained the fresh appearance of cucumber. On the same study, fresh-cut broccoli’s bright green color was preserved with the combination of melatonin and chitosan, while the incorporation of 100 mg L^−1^ melatonin caused increased microbial spoilage and chlorophyll losses (yellowing) [27]. In another study, Viacava et al. [13] mentioned that when thyme EO applied on fresh-cut lettuce resulted to lower quality product (visual quality, browning, texture, color, and odor) compared to the microencapsulated EO in which no off-odors or severe browning were observed. The application of whey permeate (0.5%, 1.5%, and 3%) on fresh-cut lettuce and carrots did not affect negatively sensory attributes of lettuce, while the highest concentration resulted to higher surface whitening on carrots [11]. Notably, Deza-Durand and Petersen [51] reported that even cutting direction (transverse cutting) during processing of fresh-cut lettuce can influence aroma development and respiration rate due to severe tissue damage. These findings suggest that losses of fresh produce quality attributes may arise from processing, applied products (concentration, time of application, and source), and the product itself.

Postharvest weight loss due to moisture loss can cause deterioration and shrinking of leaf tissue [52]. In this study, up to 7.51% weight loss was observed with the application of AA, while chitosan and Chitosan+AA resulted to lower weight losses. Chitosan application to a plant surface (via dipping or spraying) can form an edible coating that can act as a gas barrier (O_2_ and CO_2_) exchange and slow respiration rate [28,53]. It has also been suggested that edible coatings when applied to fresh produce might prevent moisture losses [53], and the combination of edible coating material with hydrophobic and hydrophilic properties is preferred on postharvest applications.

Among fresh produce, lettuce consists a commodity with moderate respiration rates and tissue wounding (due to mishandling or processing) might accelerate its respiration rate [51]. Increased respiration rate was observed when the combinations of EO+AA and Chitosan+AA were applied, while chitosan applied alone resulted to lower respiration rates. These findings are in agreement with Romanazzi et al. [28] who suggested that use of chitosan on fruits and vegetables (film formation) can reduce respiration rate acting as a gas exchange barrier, but not as water barrier due its hydrophilic properties. Mechanical damage of leafy vegetables due to cutting, storage, and transport might induce ethylene release, which in combination with increased weight loss can result in fibrosis and lignification on cut surfaces [4,54]. In this study, the application of marjoram EO decreased ethylene emission, while increased ethylene levels were reported with the application of EO+Chitosan. It has been suggested that a physiological disorder like russet spotting on lettuce can be induced by exposure to ethylene [55]. This might explain why the application of EO preserved the visual quality of fresh-cut lettuce and at the same time decreased its ethylene emission in our study. Ethylene has been linked with the induced production of plant defense compounds, such as antioxidants and phenols, as well as with the activation of enzymes including chitinase, peroxidase, phenylalanine ammonia-lyase, and polyphenol oxidase [56]. It has also been mentioned that the production of ethylene in combination with moisture loss will result in fibrosis and lignification of cut tissue, which will eventually lead to aging, senescence, and quality losses of processed lettuce [4]. Previous indications have shown that lettuce produces very little ethylene and at the same time it is sensitive to the presence of ethylene [3]. Interestingly, Hamanaka and Izumi [57] examining the effects of mustard and hop extracts on shredded cabbage and sliced cucumber reported lower respiration rates and ethylene production in both products.

Among fresh produce quality attribute, color, and appearance are the most important ones, as they are the first attributes perceived by consumers and can affect their buying decision [58]. The degree of browning in vegetables can be assessed using the L*, a*, and b* chromaticity coordinates. As previously mentioned by Chen et al. [4], browning might occur with lower L* and higher a* and b* values. Decreased L* values were observed with the use of chitosan and Chitosan+AA, while high a*, b*, and hue values were also noted suggesting a darker (less green) leaf and the formation of brown pigments. Similarly, CI and chroma values decreased with the application of chitosan and its combinations (EO+Chitosan, Chitosan+AA) and at the same time increased browning index was observed with chitosan. All these results indicated the development and the degradation of lettuce visual quality. The application of AA increased WI of fresh-cut lettuce. Chen et al. [4] reported that the application of clove essential oil (0.05%) and eugenol (0.05%) on fresh-cut lettuce maintained its bright green color during 12 days of storage at 4 °C with high L* and b* values, as well as lower a* values. On the same study, lower browning index values were also found after the use of clove EO and eugenol, compared to the 5% ethanol treatment [4]. In another study by Martin-Diana et al. [11], they reported that 3% whey permeate applied on fresh-cut lettuce resulted in high a* values compared to lower concentrations; however, b* value, hue, and chroma value did not vary. Viacava et al. [13] reported that thyme EO treatment resulted in lower L* values, while the microencapsulation of thyme EO did not affect negatively fresh-cut lettuce green color throughout storage (up to 12 days) at 5 °C, suggesting that the combination of film forming compounds can enhance/improve EOs attributes.

Total soluble solids were reduced on the second and fourth day of storage with chitosan application. As reported previously by Moreira et al. [59], the decrease in TSS might be associated with respiration rate and the consumption of sugars during this process. In the present study little to no relation between respiration rate and TSS of fresh-cut lettuce was observed, as respiration rate slightly increased on the second day, while it significantly decreased on the fourth day of storage with the application of chitosan. Lettuce’s total acidity increased with EO and chlorine application after four days stored at 7 °C, whereas sweetness decreased with the application of chlorine on the fourth and sixth day of storage. According to Zhao et al. [27], treatment with melatonin incorporated in a chitosan based bilayer coating (2.5% *w/v*) maintained the sugar–acid ratio (ripening index-sweetness) of fresh cut cucumber. In a previous study, the application of marjoram EO+AA and marjoram Hydrosol+AA increased shredded carrots TSS compared to the hydrosol when applied alone, while increased TA was reported with the application of AA [15]. It seems that when natural products are applied on minimal processed fresh produce, they can differently affect quality parameters depending on their properties, the time of application or the product they are applied on, and tailor-made investigations are needed for each commodity.

One of the main issues in fresh-cut vegetable processing is the browning of the cut surface which is an undesirable feature leading to reduced marketability products (darkening and softening), loss of nutritional value, and the development of off-flavors [21,46]. Browning results from enzymatic and non-enzymatic reactions that are favored by the disruption of plant cell wall during processing and the exposure of phenolic compounds to oxygen and enzymes [18,60]. The effects of natural products as anti-browning agents applied on fresh produce have been previously assessed [4,18,21,46,61]. The application of oxalic and ascorbic acid on lettuce aliquots maintained phenolic content on high levels compared to citric acid and cysteine application [18]. In this study, the application of EO+AA and Chitosan+AA combinations increased lettuce’s phenolic content after four and six days of storage. Interestingly, in a study by Viacava et al. [13] it has been shown that the application of thyme EO on minimally processed lettuce increased total phenolics, total flavonoids, and antioxidant capacity. Ascorbic acid and carotenoid content were increased with the use of EO+AA. According to Altunkaya and Gökmen [18], color changes have been linked with the degradation of ascorbic acid and this might explain the positive effects of EO and EO+AA on the color of fresh-cut lettuce throughout storage time. Interestingly, fresh-cut lettuce’s chlorophylls decreased during storage, though the application of Chitosan+AA and EO maintained their levels up to the last day of storage. Another study reported that high green-tea extract concentration (up to 1% *w/v*) prevented ascorbic acid and carotenoid loss of fresh-cut lettuce [61].

Antioxidant activity of fresh-cut lettuce increased with the application of EO+AA. It has been shown that AA can react and scavenge radicals and regenerate polyphenols after their oxidation, due to its antioxidant activity [18,62]. Furthermore, EOs possess antioxidant activity among others, thus when applied alone or in combination with AA might have resulted to higher phenolic content and antioxidant levels when applied to fresh-cut lettuce. In a previous study, it has been shown that the application of spearmint and lavender EO and their mixture on endive leaves at concentrations as low as 0.001% resulted in lower antioxidant capacity compared to higher concentrations (up to 0.1%) where decreased antioxidants were reported [49]. As it has been previously mentioned by Kang and Saltveit [63], wounding of plant tissue can result in increased antioxidant activity in romaine and iceberg lettuce. The application of eugenol, carvacrol, and trans-anethole on cellulose sachets resulted in higher phenolic content as well as antioxidant capacity of fresh-cut iceberg lettuce when exposed to them [64]. These findings suggest that the applied processing methods might induce defense mechanisms of plant tissue (i.e., increase in antioxidant capacity) that will lead to rapid deterioration and quality losses. This seems to be associated with the applied method, the time of application, the processing product, and the storage conditions, among other parameters.

The production of active oxygen species (i.e., H_2_O_2_, superoxide-O^2•-^, hydroxyl-OH^•^, singlet oxygen-^1^O_2_) has been linked with lipid peroxidation, production of brown pigments and core browning, pigment bleaching (chlorophyll and carotenoids), and other processes that lower postharvest quality of fruit and vegetables [56,65,66,67]. In this study, H_2_O_2_ was increased with the use of EO, AA, and their combination (EO+AA) on the second day, while decreased H_2_O_2_ levels were revealed with the use of Chitosan+AA at the end of storage. Increased lipid peroxidation was also found with Chitosan+AA on the second day, whereas EO application showed lower MDA levels after six day of storage at 7 °C. An increase in H_2_O_2_ and MDA levels was reported during storage of fresh-cut rocket and melon at 4 and 20 °C with MDA levels lower than 0.3 nmol g^−1^ fresh weight, which suggested a moderate lipid peroxidation level according to Cavaiuolo et al. [68]. Noticeably, Ferrante et al. [69] reported higher lipid peroxidation values on fresh-cut lamb’s lettuce leaves compared to intact ones, when stored at 4 °C up to 8 d (values up to 0.041–0.051 nmol kg^−1^ MDA), confirming that processing (i.e., cutting) induces plant stress and increases lipid peroxidation that utterly result to low quality products presenting postharvest disorders such as tissue browning and leaf yellowing. Additionally, in another study the use of AA when combined with marjoram EO and hydrosol increased the H_2_O_2_ and MDA levels of shredded carrots suggesting that applied treatments might also induce oxidative stress and affect the quality of the end product [15].

Fresh-cut lettuce is prone to enzymatic browning and has been associated with the activity of three enzymes: polyphenol oxidase, peroxidase, and phenylalanine ammonia lyase-PAL [46]. The use of natural products, i.e., ascorbic acid and EOs as anti-browning agents has been previously studied and results are promising [4,46,70,71]. Chen et al. [4] reported that the application of 0.05% clove EO and eugenol (used alone) decreased fresh-cut lettuce browning by significantly inhibiting the activities of PAL, PPO, and POD. The use of phytoncide (EO derived from pine leaves) (1% diluted in 95% ethanol) was able to decrease the activity of PPO, POD, and PAL of fresh-cut lettuce stored at 12 days at 4 °C. In our study, EO+AA and EO+Chitosan decreased POD activity on the fourth day of storage, while EO, EO+Chitosan, EO+AA, and Chitosan+AA also decreased POD activity on the last day of storage. The application of AA in the present study was found to increase CAT activity, while Chitosan+AA decreased POD and increased PPO activity. It has been suggested that AA does not interact directly with PPO, as it can reduce o-quinones (enzymatically produced) to their precursor diphenols [72]. A study by Li et al. [73] showed that polysaccharide-based edible coatings (alginate, chitosan, and carrageenan) are able to inhibit fresh-cut lettuce enzymatic browning by maintaining total phenols content as well as decreasing PPO and PAL activities throughout storage. In the same study, it was suggested that among the studied edible coatings, chitosan (1%) was the most effective [73]. Interestingly, increased SOD and POD, and at the same time decreased PPO activity, was reported throughout storage (up to 15 days at 4 °C), with the application of 1% chitosan on cut lettuce [73]. In our study, the combination of Chitosan+AA and Chitosan+EO increased PPO activity during the second and fourth day of storage, respectively. Moreover, the findings in our study suggest that the Chitosan+AA increased SOD activity on the second day, while when chitosan was applied alone resulted in decreased SOD and CAT activity on the fourth day of storage.

Many studies have assessed the antimicrobial effects of EOs on the microbial load of fresh produce [13,14,15,24], indicating the importance of safe and high-quality produce for the consumer. For instance, the application of thyme EO on minimally processed lettuce exhibited high bacteriostatic activity against mesophilic and psychtotrophic bacteria, *Enterobacteriaceae*, and yeast and molds counts [13]. In another study, thyme and tea tree EOs used alone or in combination resulted to great reduction of *Escherichia coli* O157:H7 population (up to 2 log) inoculated on fresh lettuce leaves stored at 10 °C [74]. Bagamboula et al. [24] reported that thyme and basil EOs at the concentrations applied (0.5 and 1%) were able to reduce both inoculated (*Shigella sonnei* and *Shigella flexneri*) and indigenous flora populations on fresh-cut lettuce (up to 2 log reduction). On the other hand, one of the main activities chitosan exhibits when applied to plants is its antimicrobial activity, and it has been previously assessed by researchers against a wide range of microorganisms [28]. Goñi et al. [75] reported that the preharvest application of chitosan (0.1% *w/v*) during the last development stages controlled lettuce’s native microflora, as well as reduced *E. coli* survival inoculated on the edible plant parts. Another study reported lower microbial load of total bacteria and the presence of *Salmonella* in ready-to eat lettuce when treated with allyl- and benzyl-isothiocyanates and chitosan [76]. In the present study, chitosan decreased lettuce’s TVC and yeast and mold counts after six days of storage at 7 °C. In addition, EO, along with EO+Chitosan, EO+AA, and Chitosan+AA, resulted in lower yeast and molds counts on the fourth day of storage, highlighting the antimicrobial activities of the examined natural compounds. These results are in accordance with studies that suggest that when chitosan is applied alone or in combination with antimicrobial compounds can inhibit microbial growth [32,77]. Noticeably, the combination of citric and ascorbic acid was not able to remove *E. coli* and *Listeria monocytogenes* cells from lettuce leaves and prevent biofilm formation [78]. However, the application of AA (1%) alone or in combination with marjoram EO and hydrosol resulted in the reduction of TVC and yeast and molds counts of shredded carrots stored at 4 °C for up to nine days [15]. Akbas and Ölmez [16] also reported that dipping of inoculated iceberg lettuce leaves on ascorbic acid solutions (0.5 and 1 %) were able to reduce *E. coli* population by almost 2 log and *L. monocytogenes* by 1 log.

## 5. Conclusions

The natural-based products used in the current study (marjoram EO, AA, and chitosan) and the outcomes of their application on fresh-cut lettuce provide encouraging evidence for the use of natural compounds in the food industry. Marjoram EO improved aroma and visual quality of lightly processed lettuce, while chitosan used alone negatively affected lettuce color. The combination of EO+AA increased phenolic, ascorbic, and carotenoid contend as well as the antioxidant status of fresh-cut lettuce, thus improving its nutritional value. Interestingly, chitosan, EO, EO+Chitosan, and Chitosan+AA presented antimicrobial activity against TVC and yeast and molds counts of minimally processed lettuce. The findings of this study are promising as the combination of natural products with antioxidant and antimicrobial activities is gaining interest for the maintenance of postharvest quality of fresh commodities and lightly processed vegetables. EO encapsulation could possibly be considered in future studies in order to increase the EO effectiveness during postharvest applications.

## Data Availability

Not applicable.

## Supplementary Materials

The following are available online at https://www.mdpi.com/2304-8158/10/3/575/s1, Figure S1: Application of chitosan (0.5–1%) on fresh-cut lettuce (preliminary test for determining the time of application: 1–5–10 min), Figure S2: Screening of marjoram essential oil (EO), chitosan (Chit) and ascorbic acid (AA) on fresh-cut lettuce weight loss (%), Figure S3: Preliminary screening of marjoram essential oil (EO), chitosan and ascorbic acid (AA) on fresh-cut lettuce color, Figure S4: Impact of marjoram essential oil (EO), chitosan, and ascorbic acid (AA) alone and their combinations on fresh-cut lettuce color.
