# Supplementary material for: The Combined and Single Effect of Marjoram Essential Oil, Ascorbic Acid, and Chitosan on Fresh-Cut Lettuce Preservation
Source: Foods. 2021 Mar 10;10(3):575. doi: 10.3390/foods10030575 (PMC8035693; doi:10.3390/foods10030575)
Supplement: Supplementary file 1 [file foods-10-00575-s001.pdf]

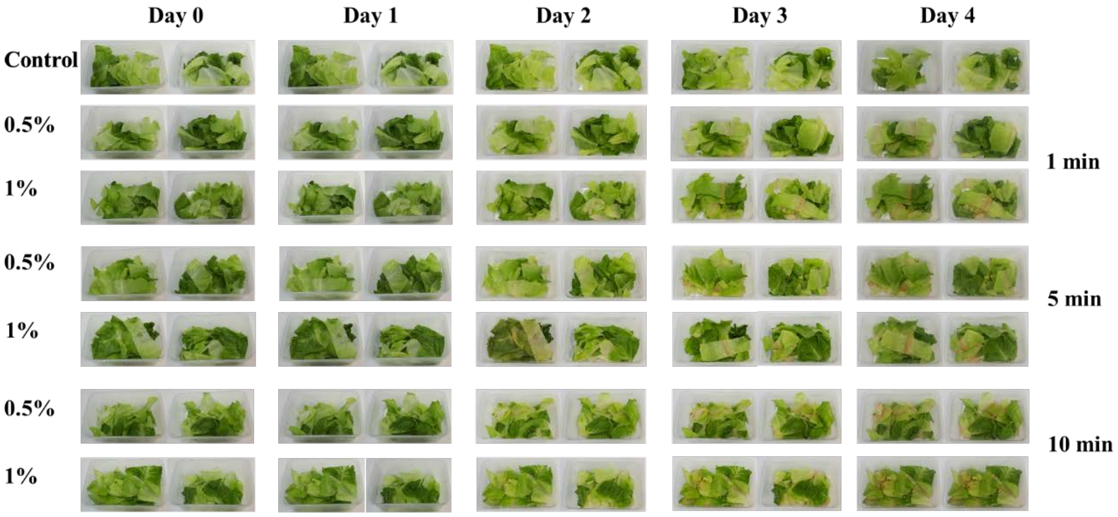

Figure S1. Application of chitosan (0.5–1%) on fresh-cut lettuce (preliminary test for determining the time of application: 1–5-10 min) and up to 4 d of storage at 7 °C and 90% RH. In each day, two representative samples for each treatment are shown.

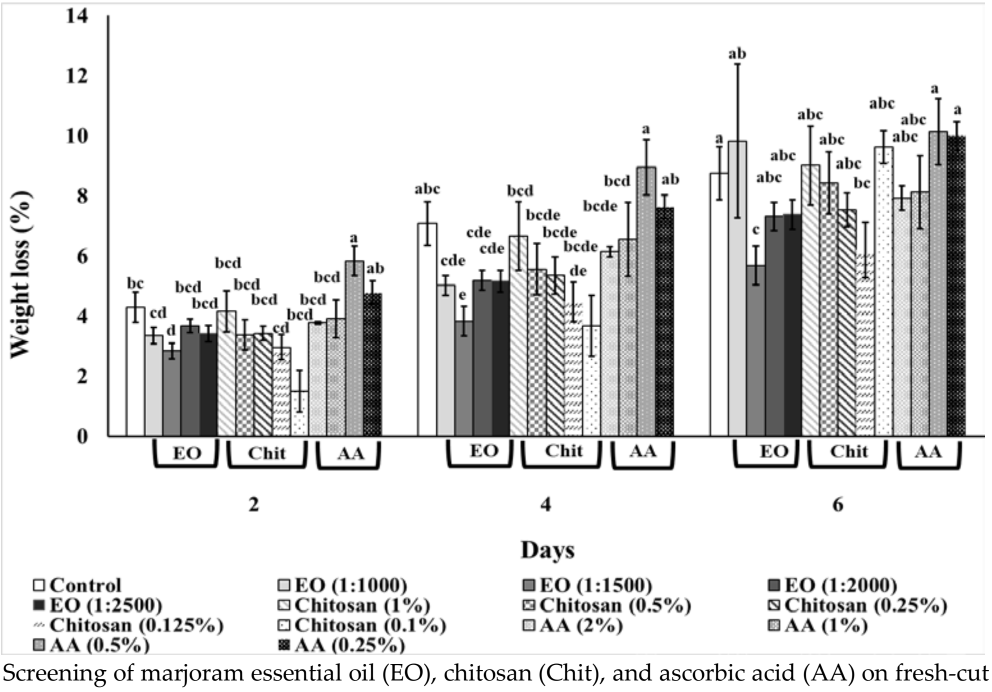

Figure S2. Screening of marjoram essential oil (EO), chitosan (Chit), and ascorbic acid (AA) on fresh-cut lettuce weight loss (%) after 6 d storage at 7 °C and 90% RH. On the columns, significant differences (P<0.05) among treatments are indicated by different Latin letters for different days. Values represent means (± SE) of measurements made on four biological replicates per treatment.

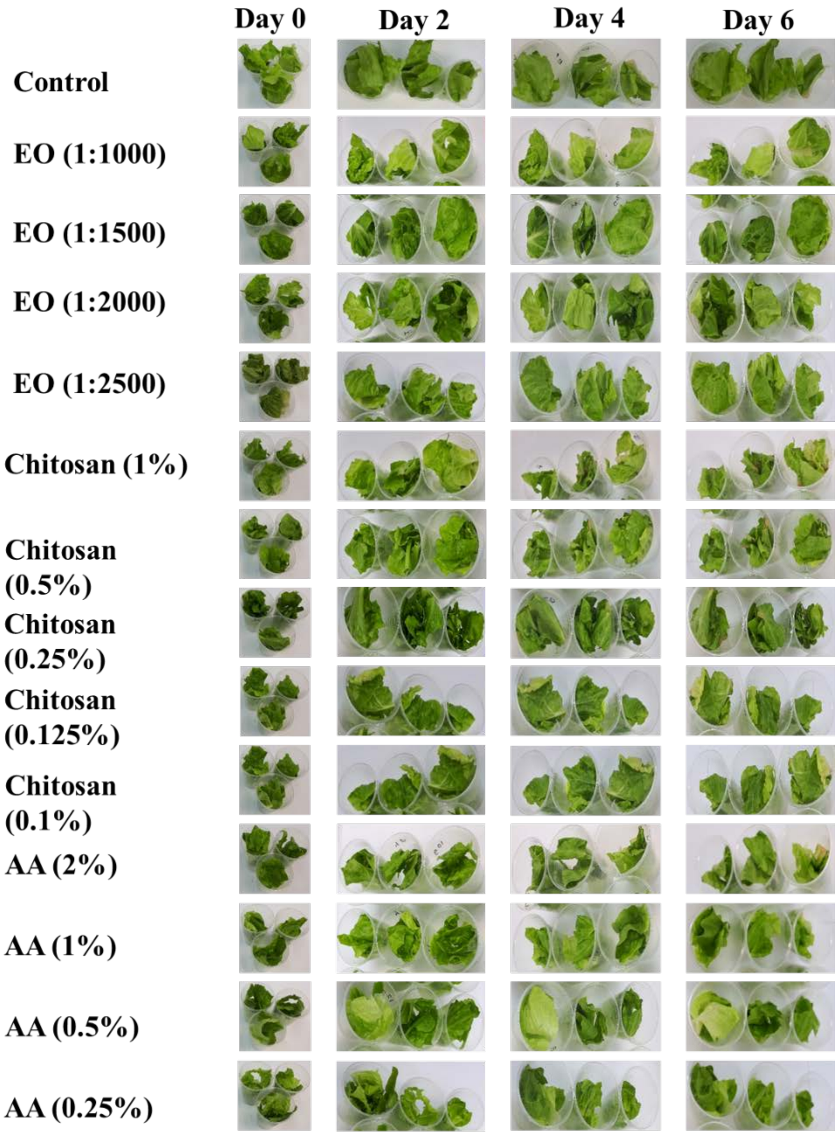

Figure S3. Preliminary screening of marjoram essential oil (EO), chitosan, and ascorbic acid (AA) on fresh-cut lettuce color after treatment and up to 6 d of storage at 7 °C and 90% RH. In each day, three representative samples for each treatment are shown.

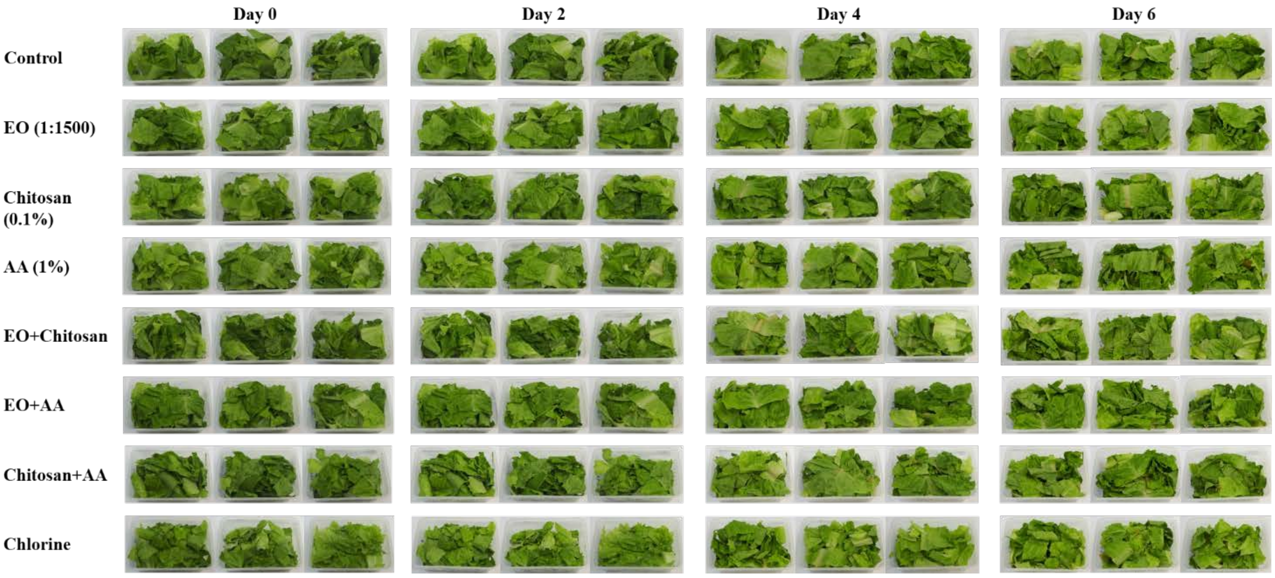

Figure S4. Impact of marjoram essential oil (EO), chitosan, and ascorbic acid (AA) alone and their combinations on fresh-cut lettuce color after treatment and up to 6 d of storage at 7 °C and 90% RH. In each day, three representative samples for each treatment are shown.
